# Antifungal activity and mechanism of *Phoebe bournei* wood essential oil against two dermatophytes

**DOI:** 10.3389/fmicb.2025.1539918

**Published:** 2025-02-07

**Authors:** Yan Yang, Qinglin Sun, Yuting Zhang, Junhua Huang, Wenjun Ma, Qi Yang, Zaikang Tong, Junhong Zhang

**Affiliations:** ^1^State Key Laboratory of Subtropical Silviculture, School of Forestry & Bio-technology, Zhejiang A&F University, Hangzhou, China; ^2^State Key Laboratory of Tree Genetics and Breeding, Research Institute of Forestry, Chinese Academy of Forestry, Beijing, China

**Keywords:** *Phoebe bournei* wood essential oil, antifungal activity, cell membrane permeability, reactive oxygen species, hub genes

## Abstract

**Background:**

Dermatophytes are notorious pathogenic fungi that threaten human health and reduce quality of life. *Phoebe bournei* (Hemsl.) Yen C. Yang wood essential oil (PWEO) has been found to have excellent antifungal activity, but its mechanism remains unclear.

**Methodology:**

Determination of minimum inhibitory concentration (MIC) of PWEO on two dermatophytes (*Microsporum gypseum* and *Epidermophyton floccosum*) by broth microdilution method. Culture plates containing PWEO in vitro tested PWEO inhibition effect of mycelial growth of dermatophytes and the effect of PWEO on hyphal structure was observed by microscopy; the changes of cell membrane permeability and the degree of lipid peroxidation were reflected by measuring cell physiological indexes. ROS and MMP probe detection of intracellular ROS and MMP change. Finally, WGCNA analysis was used to identify and verify the key genes.

**Results:**

We found that the main components of PWEO are monoterpenes and sesquiterpenoids. The PWEO had strong antifungal activity, and the MIC of PWEO against both dermatophytes was 3.600 mg/mL. PWEO significantly inhibit mycelial growth, and the inhibitory effect increases significantly with increasing concentration. When the PWEO concentration reaches 1.8mg/mL, mycelial growth is completely inhibited. Microscopic observation showed that PWEO destroy the structure of hyphae. The cell membrane permeability test indicated that the cell membrane of dermatophytes was damaged by PWEO. Cellular malondialdehyde (MDA) content was positively correlated with the concentration of PWEO, suggesting that lipid peroxidation of dermatophytes was caused by PWEO. Fluorescence microscopy images showed excessive production of ROS and disruption of MMP in dermatophytes after PWEO treatment. Physiological experiment of *M. gypseum* showed significant differences in protein extravasation, extracellular conductivity and intracellular MDA content after three hours of treatment with 0.450 mg/mL PWEO compared with the control. Five hub genes were identified by weighted gene co-expression network analysis (WGCNA), of which Long chain fatty acid CoA ligase 1 (*ACSL1*) was significantly up-regulated expressed. Meiotically up-regulated 72 (*MUG72*) and GDP-mannose transporters gene 1 (*GMT1*) were significantly down-regulated expressed after PWEO treatment, which affected the growth and reproduction of *M. gypseum*. These results suggest that PWEO can be used as natural antifungal agents for sustainable applications.

## Graphical abstract

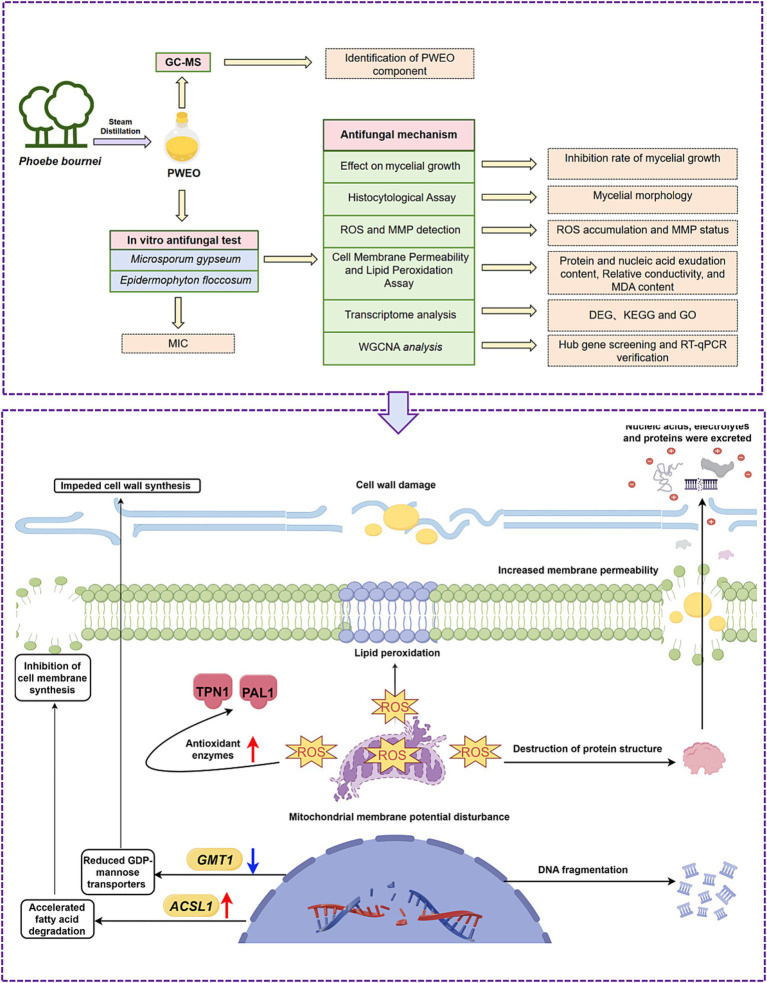

## 1 Introduction

Dermatophytes, also known as skin filamentous fungi, mainly infect the stratum corneum tissue of the host, such as hair, skin, and nails, causing cutaneous mycosis (Gottardo et al., 2023; Kim et al., 2016; Thakur and Kalsi, 2019). Cutaneous mycosis is the fourth most common disease in the world, with a global incidence of 23%, which seriously affects human life and even threatens the lives of patients with poor immune function (Maness and Zubov, 2019; Ouf et al., 2020). Traditional synthetic drugs such as ketoconazole, rezafungin, and amphotericin B are the first choice for the prevention and treatment of dermatophytosis (Forrister et al., 2024; Yuan et al., 2024; Zheng et al., 2024). However, excessive use of synthetic drugs induces dermatophyte resistance, resulting in frequent recurrence of dermatophytosis (Revie et al., 2018; Zheng et al., 2024).

In recent years, natural antifungal agents have gained increased attention in the field of healthcare and food preservation (Liu et al., 2023; Rao et al., 2019; Wang et al., 2022). Excellent natural antifungal agents have the characteristics of high efficiency, environmental sustainability, and low toxicity (Al-Abboud et al., 2024; Alamgir, 2018; Al-Rajhi and Abdel Ghany, 2023). Plants are a trove of bioactive compounds, of which essential oil is a mixture of volatile plant components. Many studies showed that plant essential oil has an excellent antifungal effect, for example, *Aloysia polystachya* EO (Areco et al., 2024), *Tetradium glabrifolium* fruit essential oil (Wang et al., 2022), and *Rosmarinus officinalis* essential oil (Bomfim et al., 2019). Therefore, plant essential oil has a significant potential for application against fungal infection.

*Phoebe bournei* is a unique important afforestation tree in China (Song et al., 2023). Its wood is fragrant and durable, and its material is dense and tough, which is an excellent material for making high-quality furniture, ships, and coffins (Han et al., 2022). With the aim of determining the inhibitory effects of PWEO on *E. floccosum* and *M. gypseum* and its antifungal mechanism, membrane permeability analysis, lipid peroxidation assay, microstructure observation, ROS generation, and MMP evaluation and related key gene mining were conducted using the PWEO.

## 2 Methodology

### 2.1 Fungal strains and culture conditions

Spore suspensions of *M. gypseum* (ATCC 42890, 10^7^CFU/mL) and *E. floccosum* (KCTC 6344, 10^7^CFU/mL) were purchased from the Shanghai Center for Preservation of Microbiology (SHMCC, Shanghai, China). The spore suspensions were diluted 10,000-fold and spread evenly on potato dextrose agar medium (PDA) at 28°C in the dark. After the activation of the fungi, mycelia were picked from PDA plates, added to potato dextrose broth (PDB) medium, and incubated at 28°C and 200 RPM for 24 h in an orbital shaker (THZ-100, Bluepard, China). Cells and supernatant harvested from PDB by centrifugation were used in physiological measurement.

### 2.2 PWEO preparation

The *P. bournei* woods with 10-year-olds were obtained from the Experimental Forest Farm (Qingyuan, China). The woods were fully crushed with a high-speed pulverizer (RS-FS1411, Royalstar, China) and passed through a 40-mesh sieve. The oil–water mixture was obtained by steam distillation, and the aqueous phase was discarded and then dehydrated with Na_2_SO_4_ (Al-Mijalli et al., 2023). A volume of 20 mg of PWEO was taken, dissolved in 1 mL of DMSO to make a 200.0 mg/mL of PWEO solution, and stored at −20°C for later use.

### 2.3 GC–MS analysis of PWEO

The GC–MS analysis for PWEO was based on the previous method with slight modifications (Al-Mijalli et al., 2023). Briefly, the analysis was carried out using a Trace GC/ISQ equipped with a TM-5 quartz capillary column (30 m, 0.25 mm × 0.25 μm), at temperatures ranging from 40°C to 280°C, with a flow rate of 3°C/min, and the temperature of the injector was set at 250°C. Helium was used as a carrier gas (1.0 mL/min), and PWEO was injected via splitless mode, with an electronic influence of 70 eV (at 200°C). The detected components were identified by comparison with the retention index (RI) of the standard compound, which was calculated based on the homolog series of n-alkanes (C7–C30). Comparing their mass spectrometry (MS) with those recorded in data libraries such as the NIST GC–MS catalogs.

### 2.4 The minimum inhibitory concentration (MIC)

The MIC of PWEO against dermatophyte was determined with reference to the methodology M38A2 of the Clinical Laboratory Standards Institute (CLSI) (Pereira et al., 2015; Kim et al., 2016). 96-well plates were prepared, and PWEO was diluted into nine mass concentrations of 7.200, 3.600, 1.800, 0.900, 0.450, 0.225, and 0.112 mg/mL in wells 3 to 9, respectively. First, 100 μL of PDB was placed in wells 3 to 9, and 200 μL of 14.400 mg/mL of PWEO was added to the second well. A volume of 100 μL of PWEO was pipetted out of the second well into the third well using the gradient dilution method, 100 μL of PWEO was pipetted out of the third well and placed into the next well, and then diluted to 8 wells in turn. The ninth well was the control group without PWEO. The 10-fold diluted 10^6^ CFU/mL of suspension was added to the 2–9 wells, and 100 μL was added to each well. Itraconazole was used as a positive control and then incubated in the incubator at 28°C for 7 days. The MIC was determined by the lowest concentration capable of visually inhibiting 100% of the fungal growth.

### 2.5 *In vitro* antifungal activities on mycelial growth

The inhibitory effect of PWEO on mycelial growth of dermatophytes was tested according to Wang et al. (2022). PDA medium with different concentrations of PWEO was prepared. PDA medium with no PWEO was set as a control check (CK), and PDA medium with DMSO as a cosolvent was set as a control group (CG). Marginal mycelial plugs (5 mm diameter) were placed on a series of plates with PDA containing different concentrations of PWEO. After incubation in a constant temperature incubator (MJX-50B, Yucheng, China) for 5 days at 28°C in darkness, the mycelia of the CK grew to approximately three-fourth of the Petri dish, and the mycelial diameter of the treated plates was measured. The inhibition rate of PWEO against *M. gypseum* and *E. floccosum* was calculated by the following formula (Xun et al., 2023):


Inhibition rate(%)=(d1−d2)/(d1−5)∗100.

where *d1* refers to the mycelial diameters (mm) on CK plates, *d2* refers to the mycelial diameters (mm) on treated plates, and 5 mm is the diameter of the mycelia plug.

### 2.6 Histocytological assay

Dermatophytes were treated with PWEO at 0.450 mg/mL for 24 h. The mycelia were collected and stained with lactic acid carbolic cotton blue staining solution (Phygene, Fujian, China) for 3 min and eluted with 75% ethanol. After temporary pack pieces were made, mycelial morphology was observed under a stereomicroscope (E5, Soptop, China).

The samples with 2.5% glutaraldehyde (XiGena, Guangdong, China) were fixed at 4°C overnight and washed three times using phosphate-buffered saline (PBS, 0.01 mol/L, pH 7.4). Afterward, the samples were dehydrated by increasing graded ethanol (50, 70, 80, 90, 95, and 100%) for 10 min at each step and then were placed in a solution containing 50, 75, and 100% tert-butanol (tert-butanol is diluted by volume with ethyl alcohol) (Liu et al., 2023; Xun et al., 2023). Each treatment lasted 20 min, with the last being air-dried. Finally, the morphology of hyphae was observed under SEM (TM4000, Hitachi, Japan).

### 2.7 ROS accumulation and MMP assay

The ROS and MMP were measured as reported previously (Xun et al., 2023). Dermatophytes were treated with PWEO at 0.450 mg/mL for 24 h and washed twice with PBS (pH 7.4). Liquid culture medium was added for resuspension, 5 mM DCFH-DA (Solarbio, Beijing, China) and 10 mM Rhodamine 123 (Solarbio, Beijing, China) solutions were added to the fungal solution, incubated at 37°C in an incubator for 30 min, centrifuged, and washed three times with PBS (0.01 mol/L, pH 7.4). The samples were then photographed using fluorescence microscopy (BX53, Olympus, Japan).

### 2.8 Cell membrane permeability and lipid peroxidation assay

The cellular leakages of proteins and nucleic acids and the conductivity of extracellular fluid were measured as reported previously (Wang et al., 2022). After a series of fungal culture solutions containing different concentrations of PWEO were incubated in an orbital shaker (THZ-100, Bluepard, China) at 200 RPM and 28°C for 24 h, cells and supernatants were collected by centrifugation at 8,000 rpm for 10 min. The conductivity of the supernatant was determined using a conductivity detector (FE38, Mettler Toledo, China), and the amount of nucleic acid exudation was determined at 260 nm using an enzyme marker (SpectraMax 190, Molecular Devices, United States). The BCA Protein Assay Kit and MDA Content Assay Kit (Solarbio, Beijing, China) were used to detect protein exudation and intracellular MDA content. Dermatophytes were treated with PWEO at 200 RPM and 28°C for 0, 3, 6, and 12 h, while dermatophytes treated without PWEO were used as CK, and the above experiment was repeated.

### 2.9 RNA extraction, library construction, and sequencing

*M. gypseum* was treated with PWEO at 0.450 mg/mL for 0, 3, 6, and 12 h, while *M. gypseum* treated without PWEO was used as CK. There were three biological replicates per treatment. 40.0 mg mycelia were collected and stored at −80°C. The total fungal RNA was extracted using a kit (DP431) (Tiangen Biotech Co., Ltd., Beijing, China). The quality control of the total RNA quantity and purity was performed using a NanoDrop ND-1000 spectrophotometer (NanoDrop, Wilmington, DE, United States), and RNA integrity was detected using a Bioanalyzer 2,100 system (Agilent, CA, United States). After the library was qualified, the Illumina NovaSeq™ 6,000 (LC-Bio Technology Co., Ltd., Hangzhou, China) was used for transcriptome sequencing, and the sequencing read length was 2 × 150 bp (PE150).

### 2.10 Identification of DEGs and functional enrichment analysis of DEGs

The gene expression level was further normalized using the Fragments Per Kilobase of transcript per Million fragments mapped reads method to eliminate the influence of different gene lengths and amount of sequencing data on the calculation of gene expression. LC-Bio Technologies online website edgeR package^1^ was used to identify DEGs with fold changes ≥2.6 and a false discovery rate-adjusted P (q-value) < 0.01. The GO analysis and KEGG analysis of DEGs were performed using the LC-Bio online cloud tool (see text footnote 1).

### 2.11 WGCNA analysis

Using the LC-Bio Technologies online website WGCNA package (see text footnote 1), the gene expression network of *M. gypseum* after PWEO treatment was constructed, and the weighted co-expression network (soft threshold R^2^ = 0.9) was constructed. Then, the co-expression modules that specifically responded to PWEO were screened out by correlation between modules and physiological traits of *M. gypseum*. According to the screening criteria (absolute value of gene significance >0.20; absolute value of module membership >0.80), genes with the highest connectivity in the significant modules were identified as candidate hub genes. Finally, Cytoscape v3.6.1 was used to visualize the top five hub genes.

### 2.12 RT-qPCR analysis

Total fungal RNA was extracted using a kit (DP431) (Tiangen Biotech Co., Ltd., Beijing, China), and then cDNA was synthesized by reverse transcription using the PrimeScriptTM RT Reagent Kit (TaKaRa, Tokyo, Japan) and stored at −20°C.

Primers for hub genes were designed using Primer Premier 6.0 (Supplementary Table S1). RT-qPCR was conducted on a CFX-96-well Real-Time System (Bio-Rad, United States), and the maltose-binding protein gene (*MBP*) was used as the reference gene. The experimental design was repeated three times, and the relative gene expression was calculated according to the 2−^ΔΔCt^ method.

### 2.13 Statistical analysis

All the experiments were performed with three replicates, and the results were presented as mean ± SD. The significance of the data between the two samples was analyzed using the *t*-test (*p* < 0.05). One-way analysis of variance was conducted using SPSS Statistics 25.0 (IBM-Armonk, New York, United States), and a *p*-value of <0.05 was considered statistically significant.

## 3 Results

### 3.1 Identification of PWEO component

The yield of PWEO was 1.51 ± 0.13‰. A total of 54 compounds were identified, including 38 terpenoids (19 monoterpenes and 19 sesquiterpenes), 6 aromatic compounds, 3 ester compounds, 3 aldehyde compounds, and 4 other components (Supplementary Figure S1). The components with relatively high content included agarospirol (18.81%), trans-calamenene (8.54%), 7-epi-a-eudesmol (6.53%), 1,5-menthadien-7-ol (6.26%), *α*-phellandren-8-ol (5.96%), (Z)-carveol (4.87%), ɑ,3-dimethylstyrene (4.38%), α-thujenal (4.02%), α-terpineol (3.87%), and cadaline (3.44%) (Supplementary Table S2).

### 3.2 Antifungal activity of PWEO

The PWEO has strong inhibition effects against *M. gypseum* and *E. floccosum*. With an increase in PWEO concentration, the germination of spores was more significantly inhibited. When the concentration of PWEO was 3.600 mg/mL, the growth of *M. gypseum* and *E. floccosum* was completely inhibited (Table 1). The MIC of PWEO against both dermatophytes was 3.600 mg/mL.

**Table 1:** Growth of dermatophytes treated with PWEO using the broth culture method.

	mg/mL								Itraconazole (μg/mL)
Dermatophytes	0.00000	0.112	0.225	0.450	0.900	1.800	3.600	7.200	
*M. gypseum*	++	++	++	++	++	++	−	−	4
*E. floccosum*	++	++	++	++	++	++	−	−	4

“++” indicates a large number of fungal growth; and “−” indicates no fungal growth.

### 3.3 Effect of PWEO on mycelial growth

**Figure 1:**
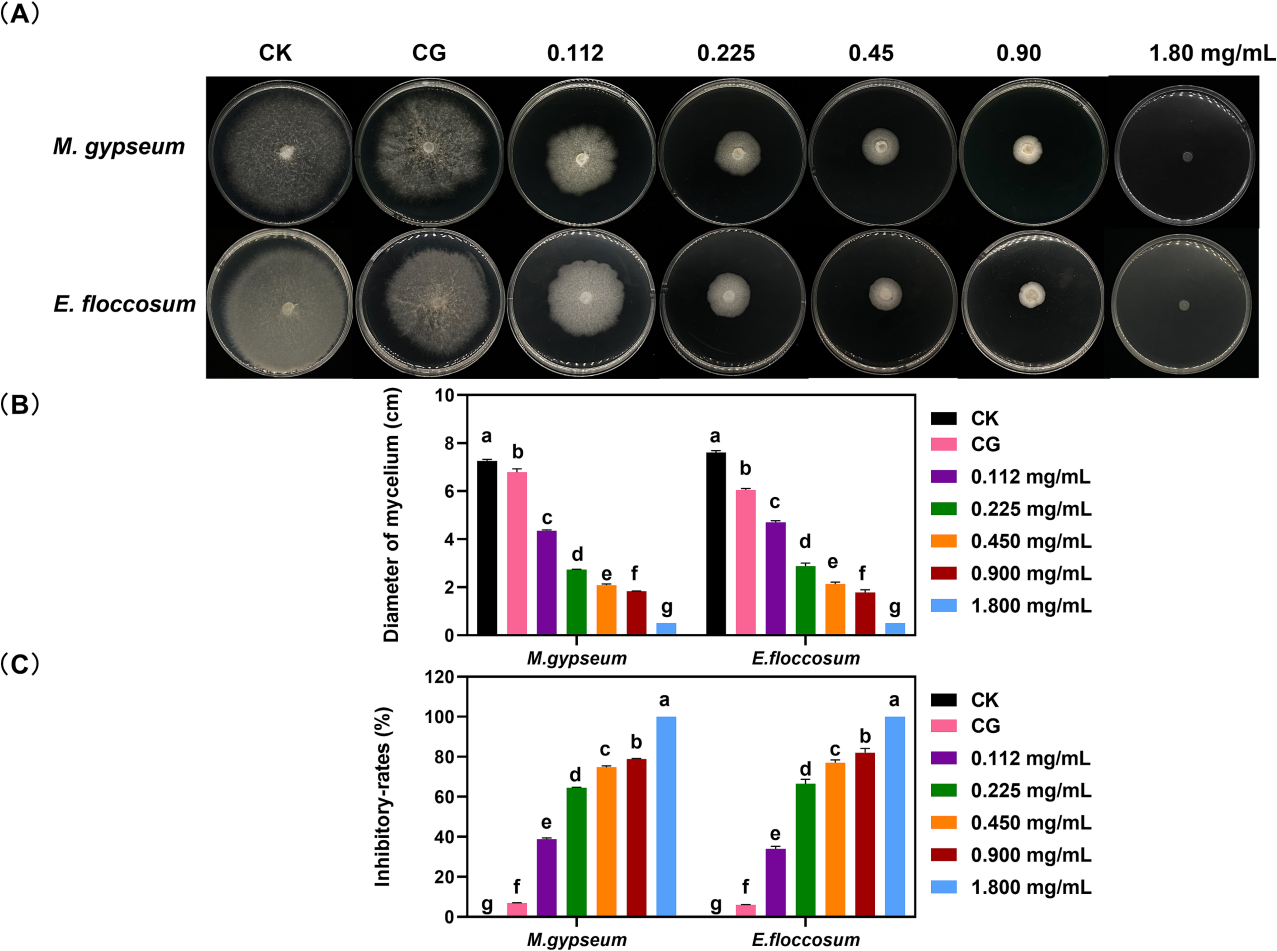
Mycelial growth status **(A)**, growth diameter **(B)**, and growth inhibitory rates **(C)** after different concentrations of PWEO treatment. Water was used as CK, and DMSO was used as CG. Data are displayed as the mean ± SD of three replications. The lowercase letters indicate significant differences (*p* < 0.05).

The PWEO inhibits mycelial growth in *M. gypseum* and *E. floccosum* growth (Figure 1A). Mycelial inhibition increases with increasing concentrations of PWEO. When the concentration of essential oil was 1.800 mg/mL, the mycelial growth of *M. gypseum* and *E. floccosum* was completely inhibited (Figures 1B,C).

### 3.4 Effects on the morphology of dermatophytes

PWEO had a strong destructive effect on the cellular structure of dermatophytes (Supplementary Figure S2). Mycelium in the CK was intact, the cell wall was smooth, the cytoplasm was abundant and uniform, and the development was normal. After 0.45 mg/mL of PWEO treatment for 24 h, mycelium became thinner, protoplast shrank, plasmolysis and cytoplasm decreased, and a large number of septa were produced. SEM showed that mycelium loses its cylindrical shape, twists, and collapses, with uneven thickness, and the surface of mycelium is rough and wrinkled after PWEO treatment.

### 3.5 Effect on cellular leakage and lipid peroxidation

**Figure 2:**
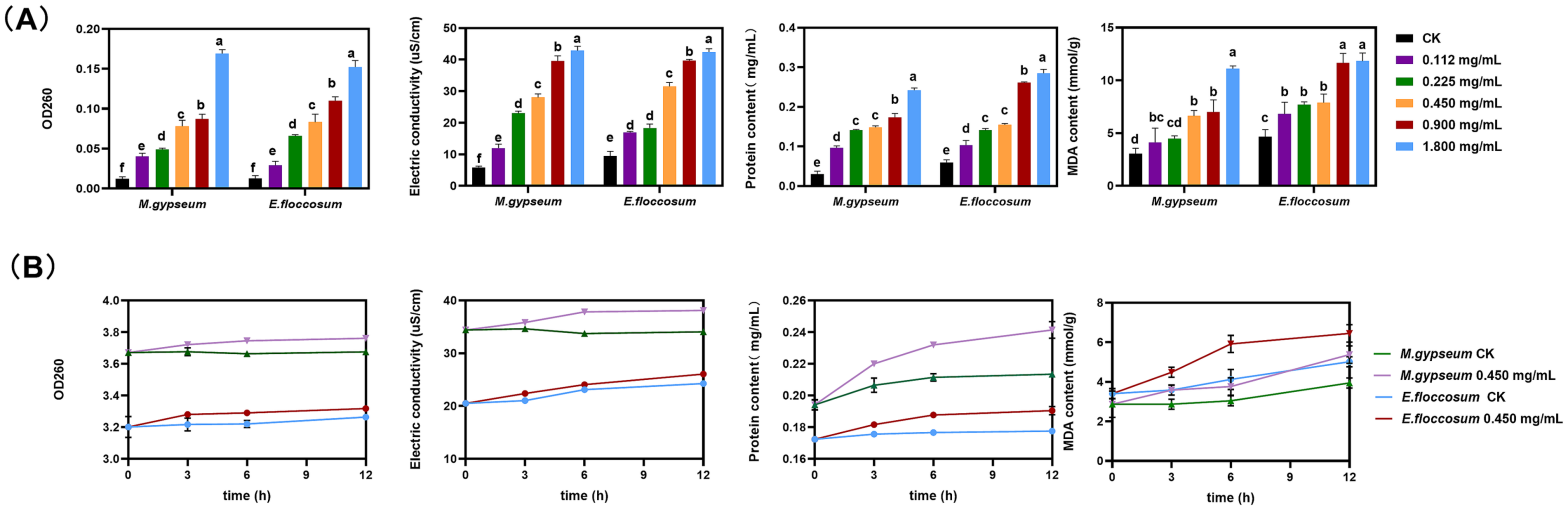
Cellular leakage and lipid peroxidation. **(A)** Effect of different concentrations of PWEO on extracellular nucleic acid content, electric conductivity, extracellular protein content, and MDA content. **(B)** Extracellular nucleic acid content, electrical conductivity, extracellular protein content, and MDA content at each time point after 0.45 mg/mL of PWEO treatment were detected. Water was used as CK. Data are displayed as the mean ± SD of three replications, and the lowercase letters indicate significant differences (*p* < 0.05).

After PWEO treatment, the extracellular nucleic acid content, electric conductivity, protein content, and the content of MDA in *M. gypseum* and *E. floccosum* cells increased significantly after PWEO treatment and were positively correlated with the concentration of PWEO (Figure 2A). For further real-time monitoring, 0.45 mg/mL of PWEO was selected. The nucleic acid content, conductivity, and protein content in the extracellular fluid stayed at a low level throughout the 12 h period in the CK. With the extension of the exposure time of 0.45 mg/mL of PWEO, the amount of nucleic acid exudation, electrolyte exudation, protein exudation, and lipid peroxidation of the two dermatophytes showed an increasing trend. After exposure to PWEO for 6 h, the amount of nucleic acid exudation of the dermatophytes was significantly different relative to CK. The conductivity, protein content of the extracellular fluid, and cellular MDA content were significantly increased after 3 h of 0.45 mg/mL of PWEO treatment (Figure 2B). These suggested that PWEO not only causes cell membrane damage and leakage of contents but also induces lipid peroxidation.

### 3.6 Effect on ROS production and MMP

Compared to the fluorescence intensity of DCFH-DA staining of mycelia, the green fluorescence was enhanced after 0.45 mg/mL of PWEO treatment, indicating that PWEO induces the production and accumulation of ROS in mycelia. MMP detection showed that green fluorescence was significantly reduced after treatment with 0.45 mg/mL of PWEO (Supplementary Figure S3).

### 3.7 RNA sequencing, identification, and functional enrichment analysis of DEGs

A total of 3.3 × 10^7^ ~ 4.8 × 10^7^ valid reads were obtained (Supplementary Table S3). PCA analysis showed a similarity between samples, and the results were biologically reproducible and reasonable (Supplementary Figure S4). A total of 5,566 DEGs were obtained after 3 h of PWEO treatment, including 2,933 upregulated genes (52.69%) and 2,633 downregulated genes (47.31%) (Supplementary Figure S5A); 4,892 DEGs were obtained after 6 h, including 3,147 upregulated genes (64.33%) and 1745 downregulated genes (35.67%) (Supplementary Figure S5B); and 5,345 DEGs were obtained at 12 h, including 3,135 upregulated genes (58.65%) and 2,210 downregulated genes (41.34%) (Supplementary Figure S5C). DEGs were mainly involved in cellular components (GO: 0005575), molecular functions (GO: 0003674), and biological processes (GO: 0008150) at each time point (Supplementary Figure S6). Analysis of KEGG signaling pathways showed that at 3 h, DEGs were mainly enriched in protein processing in the endoplasmic reticulum, cell cycle, and purine metabolism (Supplementary Figure S7A). At 6 h, DEGs were mainly enriched in amino sugar and nucleotide sugar metabolism, protein processing in the endoplasmic reticulum, and starch and sucrose metabolism (Supplementary Figure S7B). At 12 h, DEGs were mainly enriched in amino sugar and nucleotide sugar metabolism, glycerophospholipid metabolism, and protein processing in the endoplasmic reticulum (Supplementary Figure S7C). In conclusion, together, these results identify biological processes and aberrant signaling pathways involved after essential oil treatment.

### 3.8 Co-expression network construction and hub gene identification of *M. gypseum*

**Figure 3:**
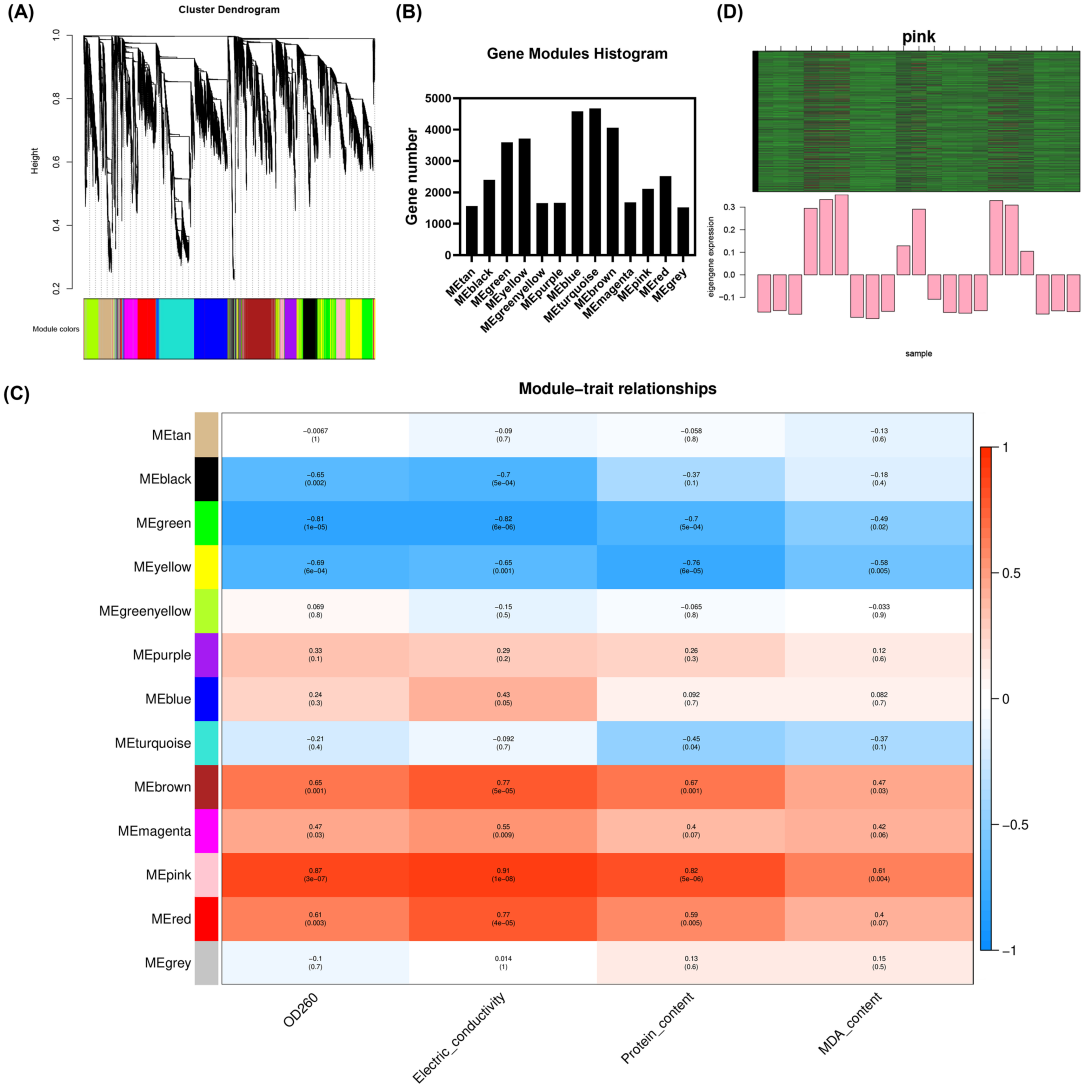
Gene modules identified by WGCNA. **(A)** Cluster dendrogram of gene co-expression networks in *M. gypseum*; **(B)** gene number of each module; **(C)** heat map of association between gene co-expression network modules and physiological indices in *M. gypseum*; and **(D)** heat map and bar chart of key gene expression in the pink module.

**Figure 4:**
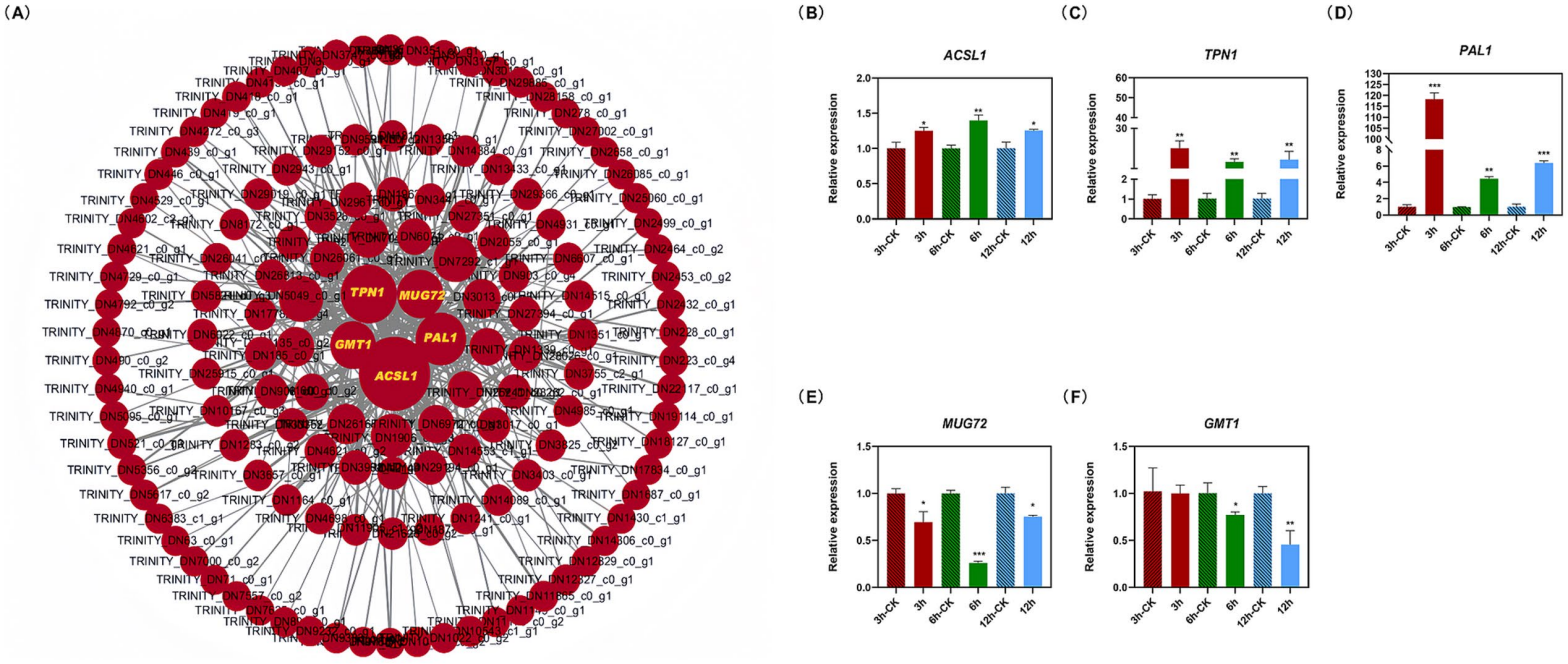
Gene interaction network map of candidate hub genes **(A)** and the relative expression of hub genes **(B–F)**. Cytoscape v3.6.1 was used to construct the gene interaction network, resulting in five hub genes including A*CSL1*, *TPN1*, *PAL1*, *MUG72*, and *GMT1*. The *MBP* was used as the reference gene. Asterisks indicate significant differences between the control and treatment (independent samples *t*-test, *p < 0.05; **p < 0.01; and ***p < 0.001).

The soft threshold of the co-expression network constructed using WGCNA was 10 (Supplementary Figure S8); modules were divided according to the hybrid dynamic shearing criterion, and the 35,776 genes were divided into 13 co-expression network modules (Figures 3A,B). The correlations between modules and physiological indexes identified the pink module including 2,110 genes (Figures 3C,D). According to the screening criteria, 407 candidate hub genes were obtained in the pink module. Cytoscape v3.6.1 was used to construct the gene interaction network, and five hub genes including *ACSL1*, *TPN1*, *PAL1*, *MUG72,* and *GMT1* were obtained (Figure 4A, Supplementary Table S4).

### 3.9 RT-qPCR validation of five hub genes

The expression levels of the *ACSL1*, *TPN1,* and *PAL1* genes were increased, while *MUG72* and *GMT1* were downregulated in *M. gypseum* treated with 0.45 mg/mL of PWEO. Specifically, the expression level of *ACSL1* reached the peak at 6 h and then decreased (Figure 4B), and the expression level of *TPN1* increased with time and reached the peak at 12 h (Figure 4C). The *PAL1* rapidly responded to the essential oil treatment at the early stage, with a rapid increase of approximately 118-fold expression, and then fell back (Figure 4D). The expression of *MUG71* decreased significantly at 6 h (Figure 4E). At the beginning of the PWEO treatment, the expression of *GMT1* did not change relative to CK but it decreased significantly over time (Figure 4F).

## 4 Discussion

At present, research on the antifungal properties of PWEO has not been found. We have determined through the microdilution of broth and *in vitro* antifungal experiments that PWEO can effectively inhibit dermatophytes with a MIC of 3.60 0 mg/mL. Previous studies have reported that the MIC of the main components of *Eucalyptus globulus* EO against *Alternaria tenuissima* is approximately 4.6 mg/mL (Singh et al., 2024). *Eucalyptus camaldulensis* EO inhibits the growth of *Aspergillus flavus*, *A. niger*, *A. terreus* and *Fusarium culmorum*, with a MIC range from 6 to 11 mg/mL (Abo Elgat et al., 2020). Rosemary EO can inhibit the growth of *Colletotrichum gloeosporioides* with a MIC of approximately 14 mg/mL (Chen, 2024). In contrast, PWEO exhibits significant antifungal activity against two dermatophytes. The components of PWEO were analyzed using GC–MS, and a large amount of monoterpenes and sesquiterpenes was found. Many terpenoids have been proven to have antifungal effects, such as thymol, caryophyllene oxide, and 1,8-cineole (Lee et al., 2023; Kamalpreet et al., 2021; Singh et al., 2024). These terpenoids may be the key components of the antifungal activity of PWEO.

Natural extracts can act against microorganisms through different mechanisms, and cell membranes are common targets (Zhang et al., 2024). Once the permeability of the cell membrane is increased, the cellular contents are extravasated in large quantities, thus affecting cell growth and proliferation. Similar to PWEO, *Atractylodes lancea* rhizomes EO (He et al., 2020), *T. glabrifolium* fruit EO (Wang et al., 2022), and allspice EO (Lee et al., 2023) can also improve the permeability of fungal cell membranes. To explain the mode of action of EO in improving cell membrane permeability, previous studies have found that EO can induce the overproduction of ROS (Lee et al., 2023; Manjinder et al., 2024), which was also confirmed in the present study by a fluorescent probe that reacted with endogenous ROS levels. Under normal physiological conditions, intracellular oxidation and antioxidant effects are in balance, but when subjected to environmental stress, the balance can be disrupted (Zadrąg-Tęcza et al., 2018). The mitochondrial membrane is an important site for bioenergy metabolism and the main site for ROS production. Excessive ROS can induce nucleic acid damage and MMP disruption (Jafri et al., 2019; Xu et al., 2022). MMP depolarizes, affecting the electron transport of the cellular electron transport chain, and eventually causes the disorder of cellular energy metabolism. ROS can also cause membrane lipid peroxidation. MDA, the final product of membrane lipid peroxidation, which was released from its production position on the membrane, can cross-link proteins and make them lose function (Cheng et al., 2011); therefore, the accumulation of MDA will aggravate membrane damage. EO has lipophilicity and can directly penetrate the lipid bilayer or directly modify the cell membrane, causing osmotic imbalance and fatty acid profile alteration (Rao et al., 2019).

In addition to directly damaging cell membranes, EO also affects membrane synthesis indirectly. We found that PWEO treatment significantly upregulated the *ACSL1*, which encodes a key enzyme for fatty acid *β*-oxidation. Fatty acids are important raw materials for synthesizing cell membranes, and the degradation of fatty acids has an undeniable impact on the synthesis of membranes. Upregulation of *ACSL1* expression in cells also promotes the incorporation of conjugated linoleic acid into neutral fat, and the increased unsaturated fatty acid content in neutral fat increases the risk of lipid peroxidation and promotes ferroptosis (Zhou et al., 2023). Therefore, *ACSL1* plays a key role in mediating lipid peroxidation, cell senescence, and apoptosis.

Vitamin B6 is phosphorylated into coenzymes, which is a cofactor of more than 180 enzymes and involved in the metabolism of enzyme systems. It is also a potential fungal ROS remover, which reduces oxidative stress in organisms and is a key molecule in stress sensitivity (Hong et al., 2023; Mascolo and Vernì, 2020). Vitamin B6 deficiency can lead to the attenuation of the antioxidant defense system and the increase of lipid peroxidation (Keles et al., 2010). *TPN1* encodes the vitamin B6 transporter, which localizes to the plasma membrane and is the only protein involved in three forms of vitamin B6 transport in yeast. *TPN1* mutants lose the ability to utilize extracellular PN, PL, and PM (Stolz and Vielreicher, 2003). The expression level of the *TPN1* gene was significantly increased after treatment with EOs, indicating that cells had increased demand for vitamin B6. Transported vitamin B6 is most likely used in cellular antioxidant processes.

Phenylalanine ammonia-lyase 1 (*PAL1*) is the rate-limiting enzyme involved in the phenylpropane metabolic pathway. PAL1 also belongs to the host defense system-related enzyme and cooperates with catalase (CAT) and peroxidase (POD) to resist the infection of pathogenic fungi; the contents of PAL1 defense enzymes in *Agaricus bisporus* were significantly increased after infection with *Mycogone perniciosa* (Zhou and Li, 2015). It has been widely studied that EO stress increases the activities of PAL1 and other antioxidant enzymes in organisms (Khumalo et al., 2017; Wang et al., 2022). Certainly, the ROS-scavenging capacity of PAL1 is limited. Under the continuous stress of EOs, the excessive production of ROS exceeds the clearance threshold of antioxidant-related enzymes, destroys the antioxidant system, makes it lose its defense function, and eventually causes irreversible cell damage.

*MUG72* encodes the meiotic upregulated gene 72 protein, whose function is relatively unknown. *However,* the *MUG66* mutant strain showed low sporulation and abnormal spore morphology, and the *MUG77* and *MUG78* mutant strains showed no spore production in their ascomycetes (Martín-Castellanos et al., 2005). Therefore, we speculate that *MUG72* may also be involved in sporulation, and further experimental studies are needed to verify its function.

Mannose-binding protein is an important component of the fungal cell wall. Mannose is also an important component of glycolipids, GPI anchors, and capsules, among which polysaccharide capsules are extremely important in the virulence of fungi (Heise et al., 2002). GDP-mannose is transported across the membrane to other organelles by GDP-mannose transporters (GMTs). Previous studies have identified two GDP-mannose transporters in *Cryptococcus*, GMT1 and GMT2, but capsule, cell wall, capsule synthesis, and protein glycosylation are more dependent on GMT1 (Kadry et al., 2018). Therefore, GDP-mannose transporter can also be used as a potential antifungal target. GMT1 may be an entry point for new antifungal agents targeting virulence determinants.

## 5 Conclusion

In the present study, we found that PWEO had potent inhibitory effects on both dermatophytes. PWEO leads to ROS overproduction, MMP disorder, lipid peroxidation, cell wall structure destruction, and increased membrane permeability, thus inhibiting fungal growth. PWEO inhibited the growth and proliferation of *M. gypseum* by upregulating *ACSL1* to promote the degradation of long-chain fatty acids and downregulating *MUG72* and *GMT1* to inhibit spore meiosis and GDP-mannose transport, respectively. These findings provide new ideas for the targeted therapy of fungal diseases. It provides a new medicinal resource for the further research and treatment of skin diseases caused by *E. floccosum* and *M. gypsum* and provides a theoretical basis for the development of new natural antifungal agents.

## Data Availability

The original contributions presented in the study are publicly available. This data can be found here: https://www.ncbi.nlm.nih.gov/sra, BioProject accession number PRJNA1217368.

## Footnotes

^1^ https://www.omicstudio.cn

## Supplementary material

SUPPLEMENTARY FIGURE S1 GC/MS chromatograms of PWEO.

SUPPLEMENTARY FIGURE S2 Morphological phenotypes of *M. gypseum* **(A)** and *E. floccosum* **(B)** observed by stereomicroscope (Scale bar = 50 μm) and scanning electronmicroscope (SEM) (Scale bar = 10 μm). Water was used as CK.

SUPPLEMENTARY FIGURE S3 Fluorescence microscopy images of ROS and MMP accumulation in *M. gypseum* **(A)** and *E. floccosum* **(B)** treated with 0.45 mg/mL PWEO. ROS stained with DCFH-DA, MMP stained with Rhodamine 123，and water was used as CK (Scale bar = 20 μm).

SUPPLEMENTARY FIGURE S4 The similarity relationship (PCA) between samples.

SUPPLEMENTARY FIGURE S5 Volcano plot of DEGs between treatment and control groups at each time point after PWEO treatment. **(A)** DEGs of 3 h; **(B)** DEGs of 6 h; **(C)** DEGs of 12 h.

SUPPLEMENTARY FIGURE S6 GO term analysis of DEG. The first lap indicates top 20 GO terms and the number of the genes corresponds to the outer lap. The second lap indicates the number of the genes in the genome background and *p* values for enrichment of the DEGs for specified biological process.

SUPPLEMENTARY FIGURE S7 KEGG enrichment analysis of DEGs between cells treated with 0.45 mg/mL PWEO for 3, 6, and 12 h and controls.

SUPPLEMENTARY FIGURE S8 Analysis of the scale-free fit index and the mean connectivity for various soft-thresholding powers. The red line indicates where the correlation coefficient is 0.9, and the corresponding soft-thresholding power is 10.

## Glossary

***ACSL1***: Long-chain fatty acid CoA ligase 1
CAT: Catalase
DEGs: Differentially expressed genes
DMSO: Dimethyl sulfoxide
EO: Essential oil
***GMT1***: GDP-mannose transporters gene 1
GO: Gene ontology
KEGG: Kyoto Encyclopedia of Genes and Genomes
MDA: Malondialdehyde
MIC: Minimum inhibitory concentration
MMP: Mitochondrial membrane potential
***MUG72***: Meiotically upregulated 72
***PAL1***: Phenylalanine ammonia-lyase 1
PBS: Phosphate-buffered saline
PDA: Potato dextrose agar medium
PDB: Potato dextrose broth medium
PL: Pyridoxal
PM: Pyridoxamine
PN: Pyridoxine
POD: Peroxidase
PWEO: *Phoebe bournei* wood essential oil
ROS: Reactive oxygen species
SEM: Scanning electron microscopy
***TPN1***: Vitamin B6 transporter 1
WGCNA: Weighted gene co-expression network analysis
