# Supplementary figures and images for: Antifungal activity and mechanism of Phoebe bournei wood essential oil against two dermatophytes
Source: Front Microbiol. 2025 Feb 7;16:1539918. doi: 10.3389/fmicb.2025.1539918 (PMC11842444; doi:10.3389/fmicb.2025.1539918)

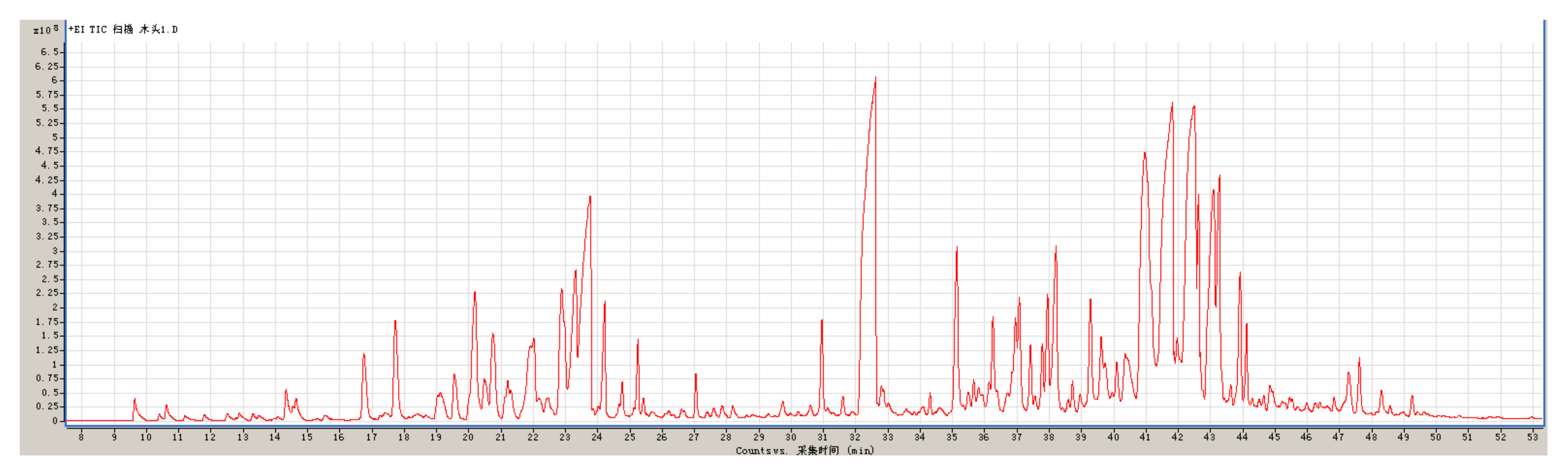

Supplement: SUPPLEMENTARY FIGURE S1 — GC/MS chromatograms of PWEO. [file Image_1.TIF]

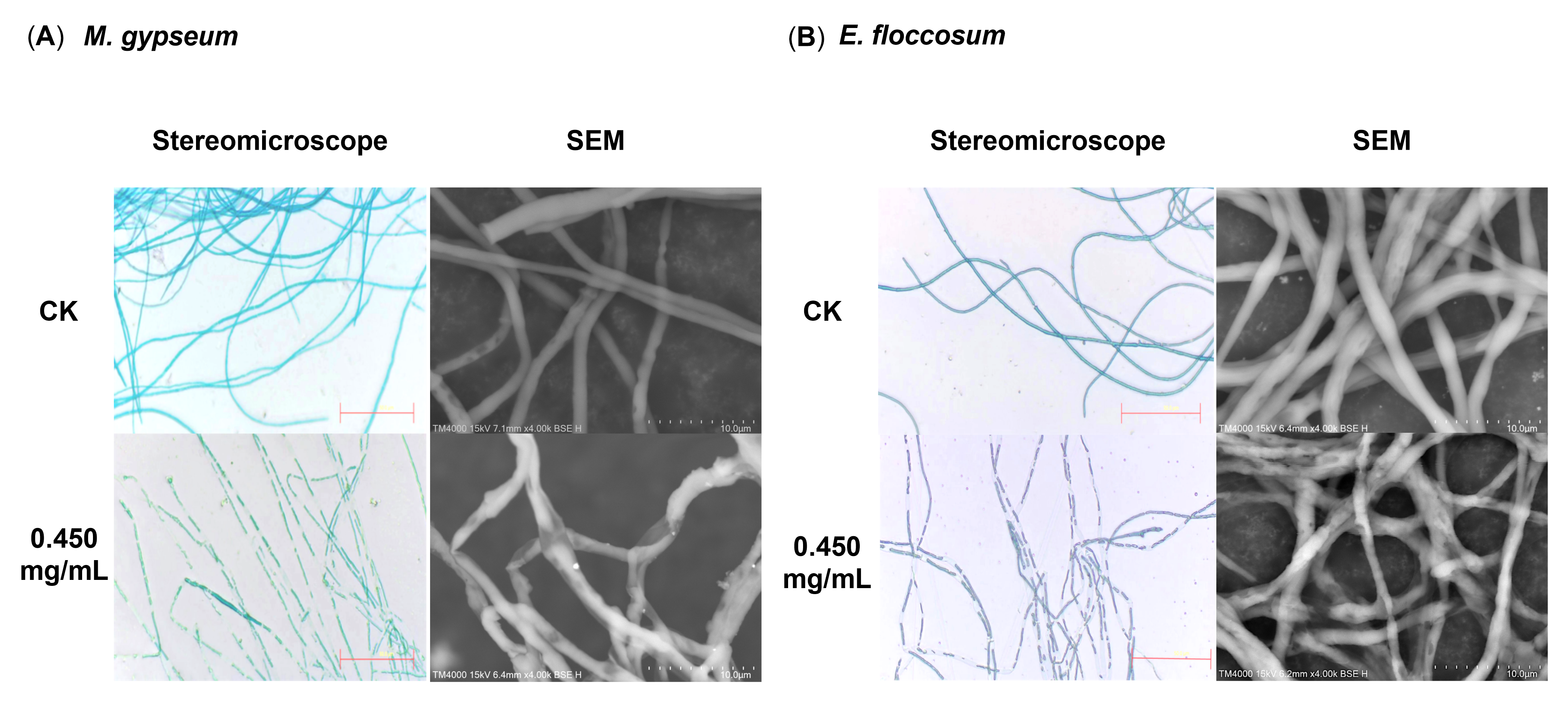

Supplement: SUPPLEMENTARY FIGURE S2 — Morphological phenotypes of M. gypseum (A) and E. floccosum (B) observed by stereomicroscope (Scale bar = 50 μm) and scanning electronmicroscope (SEM) (Scale bar = 10 μm). Water was used as CK. [file Image_2.TIF]

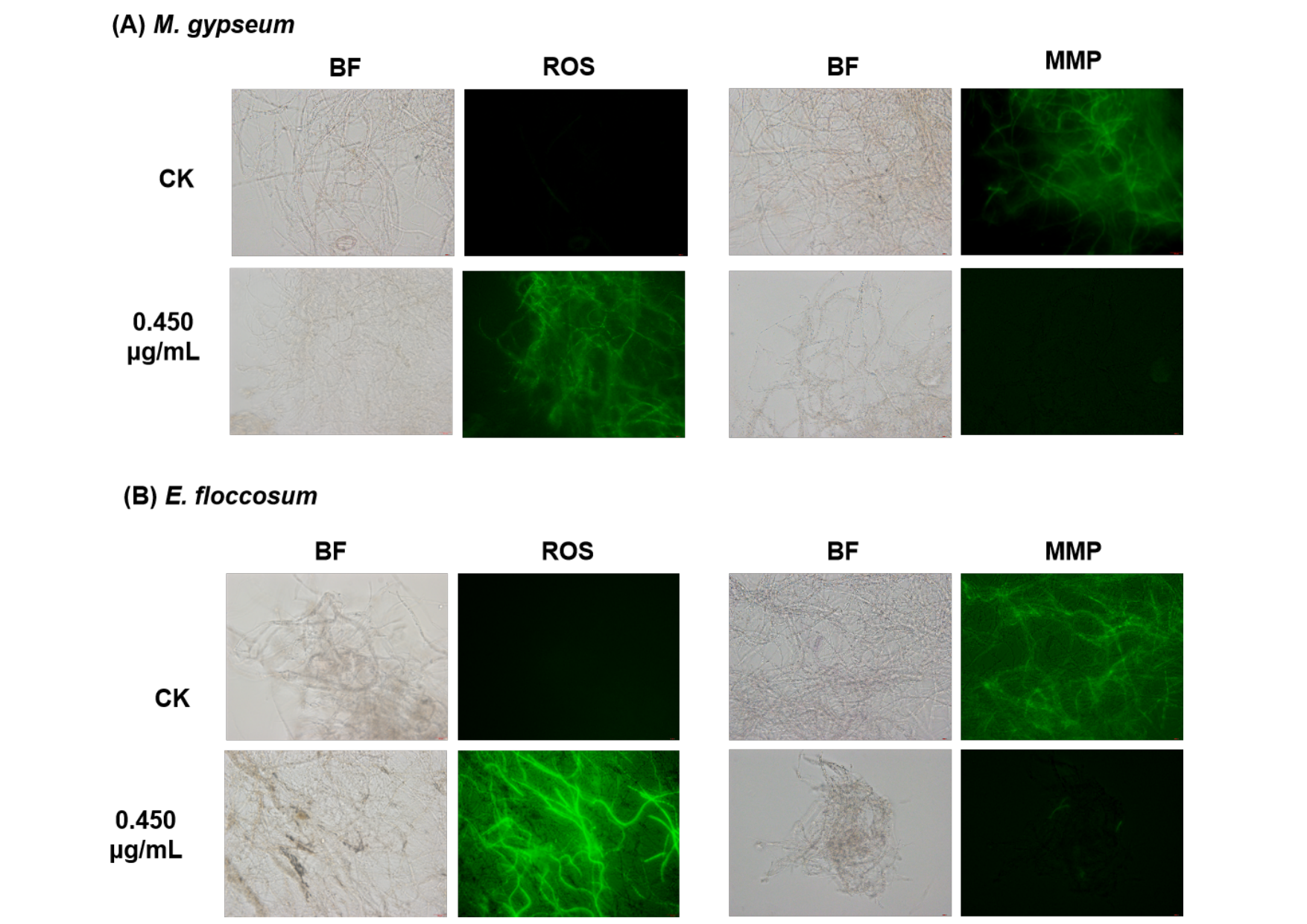

Supplement: SUPPLEMENTARY FIGURE S3 — Fluorescence microscopy images of ROS and MMP accumulation in M. gypseum (A) and E. floccosum (B) treated with 0.45 mg/mL PWEO. ROS stained with DCFH-DA, MMP stained with Rhodamine 123，and water was used as CK (Scale bar = 20 μm). [file Image_3.TIF]

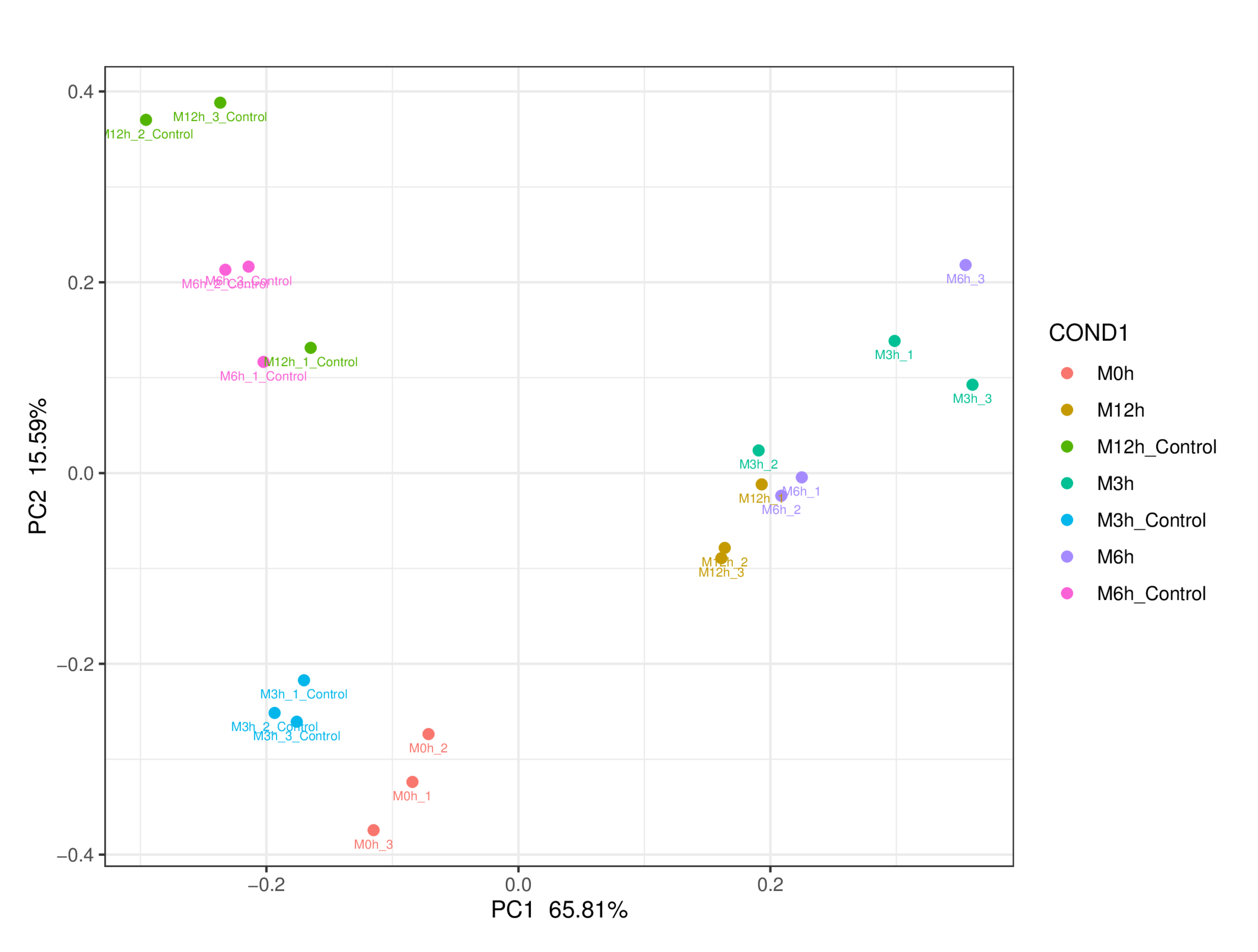

Supplement: SUPPLEMENTARY FIGURE S4 — The similarity relationship (PCA) between samples. [file Image_4.TIF]

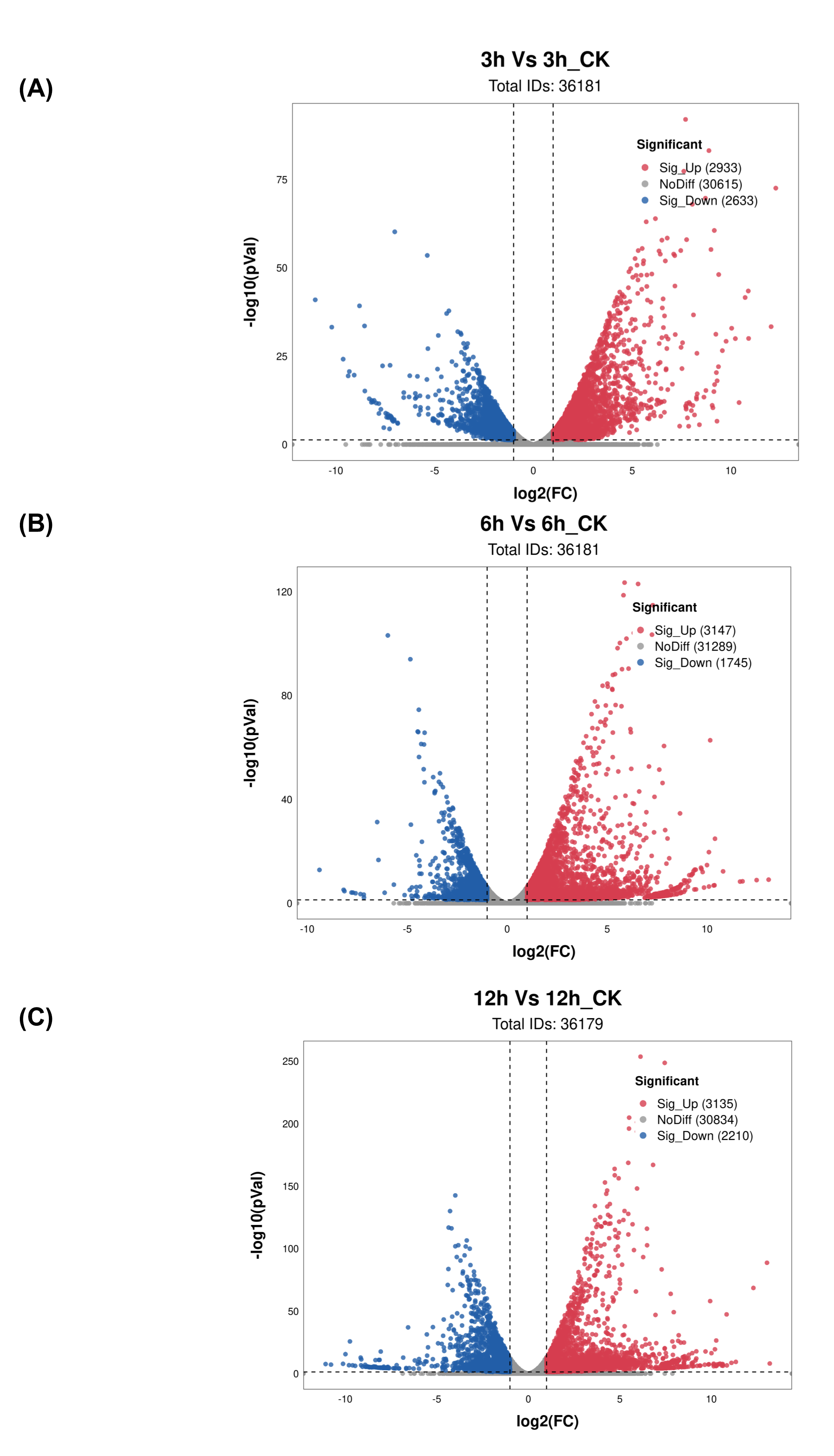

Supplement: SUPPLEMENTARY FIGURE S5 — Volcano plot of DEGs between treatment and control groups at each time point after PWEO treatment. (A) DEGs of 3 h; (B) DEGs of 6 h; (C) DEGs of 12 h. [file Image_5.TIF]

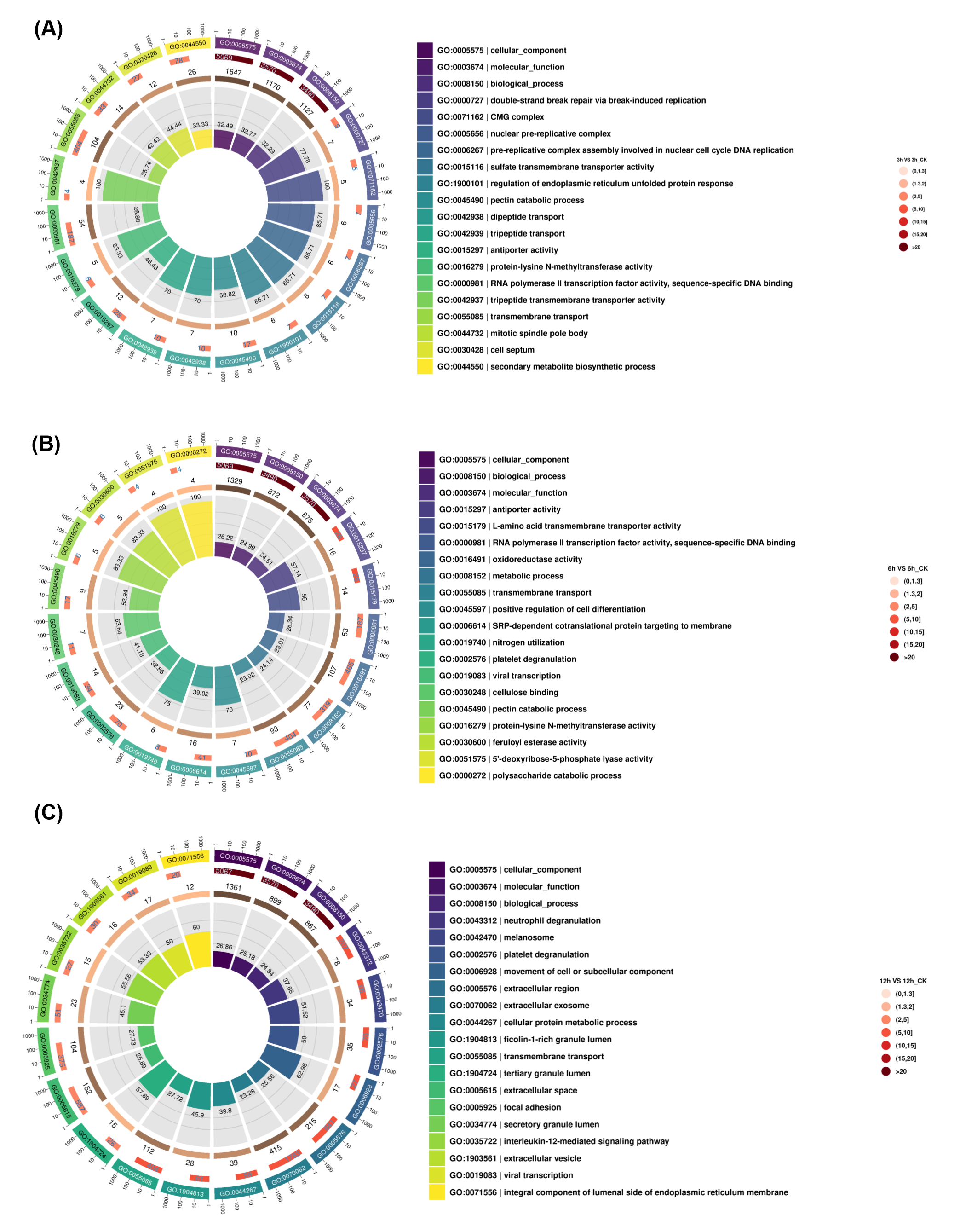

Supplement: SUPPLEMENTARY FIGURE S6 — GO term analysis of DEG. The first lap indicates top 20 GO terms and the number of the genes corresponds to the outer lap. The second lap indicates the number of the genes in the genome background and p values for enrichment of the DEGs for specified biological process. [file Image_6.TIF]

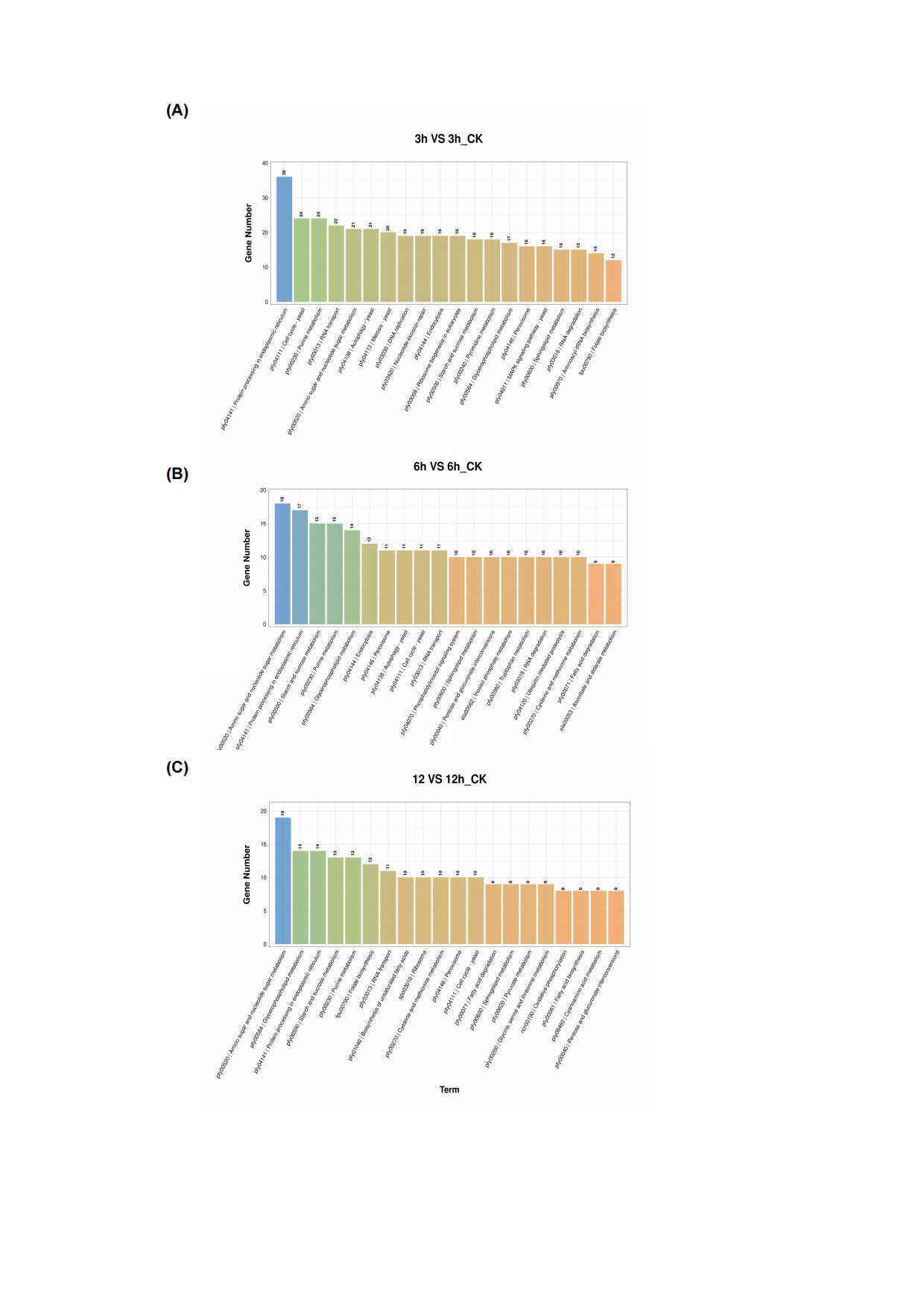

Supplement: SUPPLEMENTARY FIGURE S7 — KEGG enrichment analysis of DEGs between cells treated with 0.45 mg/mL PWEO for 3, 6, and 12 h and controls. [file Image_7.TIF]

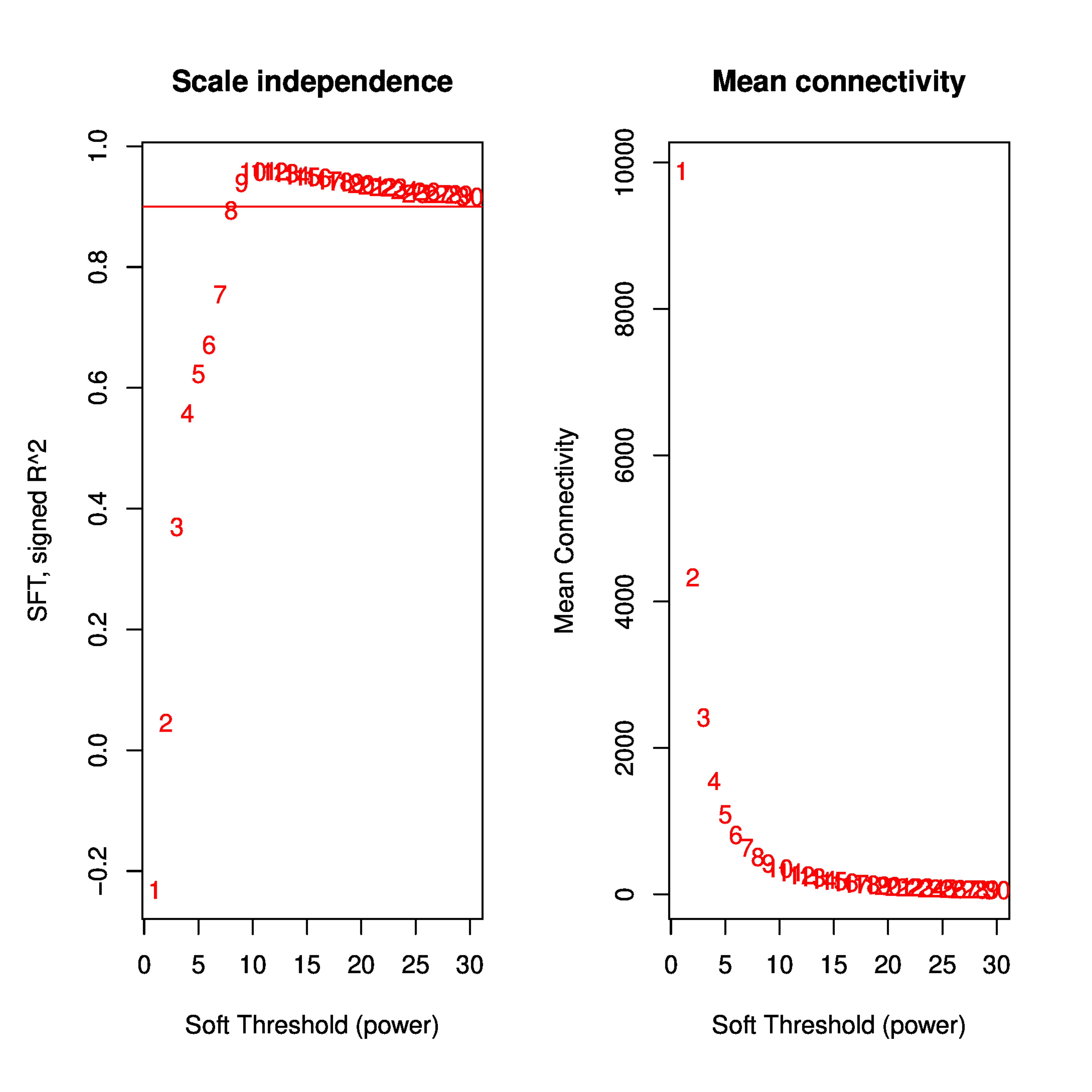

Supplement: SUPPLEMENTARY FIGURE S8 — Analysis of the scale-free fit index and the mean connectivity for various soft-thresholding powers. The red line indicates where the correlation coefficient is 0.9, and the corresponding soft-thresholding power is 10. [file Image_8.TIF]
