# Supplementary material for: Antifungal activity and mechanism of Phoebe bournei wood essential oil against two dermatophytes
Source: Front Microbiol. 2025 Feb 7;16:1539918. doi: 10.3389/fmicb.2025.1539918 (PMC11842444; doi:10.3389/fmicb.2025.1539918)
Supplement: Supplementary file 9 [file Table_1.DOCX]

| Gene name | Primer sequence |
| --- | --- |
| *ASCL1* | F: AATATACGAACAGCGCCATCG |
|  | R: CATCAAACTCCCCGATGTCAC |
| *TPN1* | F: AATATACGAACAGCGCCATCG |
|  | R: TTCTTCCTGACCACCTTC |
| *PAL1* | F: CACTGTAGATGCTGATGAC |
|  | R: GTCGGATATGAGCGGTAG |
| *MUG72* | F: GTCGGATATGAGCGGTAG |
|  | R: CAAAACGCCAACATTCCGATG |
| *GMT1* | F: ATTCGCTTTGATGGTGCTCAG |
|  | R: ATCAATGCTCTTCAAGTCGGC |
| *MBP1* | F: AGTCCTAGTTACCTTGACCG |
|  | R: CGGTGTTTAAGTGCTAGATAGG |

**Table S1.** Specific primers design for hub genes
