# Supplementary material for: Antifungal activity and mechanism of Phoebe bournei wood essential oil against two dermatophytes
Source: Front Microbiol. 2025 Feb 7;16:1539918. doi: 10.3389/fmicb.2025.1539918 (PMC11842444; doi:10.3389/fmicb.2025.1539918)
Supplement: Supplementary file 10 [file Table_2.DOCX]

| **NO.**  **Table S2.** Analysis of *Phoebe bournei* wood volatile oil components (mg/μL) | **Compounds** | **Formula** | ***P. bournei*** |
| --- | --- | --- | --- |
|  | Agarospirol | C_15_H_26_O | 0.0675 ±0.00172 |
|  | trans-Calamenene | C_15_H_22_ | 0.0307 ±0.00186 |
|  | 7-epi-a-Eudesmol | C_15_H_26_ | 0.0234 ±0.00135 |
|  | 1,5-Menthadien-7-ol | C_10_H_16_O | 0.0225 ±0.00327 |
|  | α-Phellandren-8-ol | C_10_H_16_O | 0.0214 ±0.00052 |
|  | (Z)-Carveol | C_10_H_16_O | 0.0175 ±0.00104 |
|  | ɑ,3-Dimethylstyrene | C_10_H_12_ | 0.0157 ±0.00116 |
|  | α-Thujenal | C_10_H_14_O | 0.0144 ±0.00046 |
|  | α-Terpineol | C_10_H_18_O | 0.0139 ±0.00091 |
|  | Cadaline | C_15_H_18_ | 0.0124 ±0.00045 |
|  | L-pinocarveol | C_10_H_16_O | 0.0113 ±0.00072 |
|  | D-Carvone | C_10_H_14_O | 0.0103 ±0.00076 |
|  | 3,7(11)-Eudesmadiene | C_15_H_24_ | 0.0099 ±0.00043 |
|  | Copaene | C_15_H_24_ | 0.0091 ±0.00071 |
|  | 9aβ-methano-1-benzoxepin | C_15_H_24_O | 0.0066 ±0.00033 |
|  | δ-Cadinene | C_15_H_24_ | 0.0065 ±0.00044 |
|  | trans-Verbenol | C_10_H_16_O | 0.0048 ±0.00036 |
|  | 2,4-Thujadiene | C_10_H_14_ | 0.0048 ±0.00053 |
|  | (3R,5aR,9S,9aS)-2,2,5a,9-Tetramethyloctahydro-2H-3,9a-methanobenzo[b]oxepine | C_15_H_26_O | 0.0047 ±0.00025 |
|  | Caryophyllene oxide | C_15_H_24_O | 0.0038 ±0.00018 |
|  | Thymol | C_10_H_14_O | 0.0037 ±0.00027 |
|  | Pinocarvone | C_10_H_14_O | 0.0033 ±0.00023 |
|  | Fenchol | C_10_H_18_O | 0.0030 ±0.00021 |
|  | Ethanone, 1-(4-methylphenyl)- | C_9_H_10_O | 0.0028 ±0.00039 |
|  | β-Vetivone | C_15_H_22_O | 0.0025 ±0.00007 |
|  | 1,6-dimethyl-Naphthalen | C_12_H_12_ | 0.0023 ±0.00279 |
|  | Terpinen-4-ol | C_10_H_18_O | 0.0021 ±0.00021 |
|  | (-)-Bornyl acetate | C_12_H_20_O_2_ | 0.0021 ±0.00018 |
|  | 10-Northuja-2-ene-4-one | C_9_H_12_O | 0.0021 ±0.00026 |
|  | β-Thujene | C_10_H_16_ | 0.0018 ±0.00017 |
|  | 1,3-ditert-Butylbenzene | C_14_H_22_ | 0.0018 ±0.00022 |
|  | (+)-Valencen | C_15_H_24_ | 0.0016 ±0.00013 |
|  | α-Calacorene | C_15_H_24_ | 0.0016 ±0.00009 |
|  | 4,8,8-trimethyl-2-methylene-4-vinylbicyclo[5.2.0]nonane | C_15_H_24_ | 0.0015 ±0.00022 |
|  | 3-Phenylmethacrolein | C_10_H_10_O | 0.0014 ±0.00010 |
|  | [3,5,5-trimethyl-9-methylene-7,8-dihydro-6H-benzo[7]annulene](https://china.guidechem.com/9846613/detail.html" \o "3,5,5-trimethyl-9-methylene-7,8-dihydro-6H-benzo[7]annulene" \t "https://china.guidechem.com/dict/_blank) | C_15_H_20_ | 0.0014 ±0.00010 |
|  | o-Cymene | C_10_H_14_ | 0.0012 ±0.00009 |
|  | α-Cubebene | C_15_H_24_ | 0.0010 ±0.00009 |
|  | Camphene | C_10_H_16_ | 0.0010 ±0.00010 |
|  | (-)-β-Elemene | C_15_H_24_ | 0.0010 ±0.00007 |
|  | α-Corocalene | C_15_H_20_ | 0.0009 ±0.00004 |
|  | 1,8-Cineole | C_10_H_18_O | 0.0009 ±0.00007 |
|  | Eudesma-4(14),7(11)-diene | C_15_H_24_ | 0.0009 ±0.00005 |
|  | 3-Isopropylbenzaldehyde | C_10_H_12_O | 0.0008 ±0.00006 |
|  | 10,11-Epoxycalamenene | C_15_H_20_O | 0.0008 ±0.00006 |
|  | Selina-3,7(11)-diene | C_15_H_24_ | 0.0007 ±0.00024 |
|  | Dibutyl phthalate | C_16_H_22_O_4_ | 0.0006 ±0.00004 |
|  | β-Bergamotene | C_15_H_24_ | 0.0005 ±0.00007 |
|  | 1,2,4-Metheno-1H-indene, octahydro-1,7a-dimethyl-5-(1-methylethyl)-, [1S-(1.alpha.,2.alpha.,3a.beta.,4.alpha.,5.alpha.,7a.beta.,8S*)]- | C_15_H_24_ | 0.0005 ±0.00005 |
|  | 4-Terpinenyl acetate | C_12_H_20_O_2_ | 0.0005 ±0.00002 |
|  | Dodecanal | C_12_H_24_O | 0.0005 ±0.00011 |
|  | (+)-β-selinene | C_15_H_24_ | 0.0005 ±0.00005 |
|  | Perillaldehyde | C_10_H_14_O | 0.0003 ±0.00003 |
|  | 1,2-Benzenedicarboxylic acid, bis(2-methylpropyl) ester | C_16_H_22_O_4_ | 0.0003 ±0.00001 |
