# Supplementary material for: Antifungal activity and mechanism of Phoebe bournei wood essential oil against two dermatophytes
Source: Front Microbiol. 2025 Feb 7;16:1539918. doi: 10.3389/fmicb.2025.1539918 (PMC11842444; doi:10.3389/fmicb.2025.1539918)
Supplement: Supplementary file 11 [file Table_3.DOCX]

| Sample | Raw_Reads | Raw_Bases | Valid_Reads | Valid_Bases | Valid% | Q20% | Q30% | GC% |
| --- | --- | --- | --- | --- | --- | --- | --- | --- |
| M0h_1 | 40071388 | 6.01G | 37998424 | 5.60G | 94.83 | 98.7 | 95.78 | 57.06 |
| M0h_2 | 44577298 | 6.69G | 42320382 | 6.23G | 94.94 | 98.73 | 95.88 | 57.08 |
| M0h_3 | 40867744 | 6.13G | 38923032 | 5.73G | 95.24 | 98.8 | 96.25 | 57.5 |
| M3h_1 | 38340576 | 5.75G | 36452724 | 5.37G | 95.08 | 98.81 | 96.27 | 57.43 |
| M3h_2 | 39452108 | 5.92G | 37413920 | 5.51G | 94.83 | 98.93 | 96.58 | 57.72 |
| M3h_3 | 39127886 | 5.87G | 36981252 | 5.44G | 94.51 | 98.79 | 96.2 | 57.42 |
| M3h_1_Control | 36916188 | 5.54G | 35315780 | 5.21G | 95.66 | 98.76 | 96.1 | 57.45 |
| M3h_2_Control | 40008442 | 6.00G | 37791668 | 5.56G | 94.46 | 98.76 | 96.09 | 57.52 |
| M3h_3_Control | 40338754 | 6.05G | 38405296 | 5.66G | 95.21 | 98.79 | 96.18 | 57.54 |
| M6h_1 | 51116446 | 7.67G | 47366896 | 6.93G | 92.66 | 98.11 | 94.39 | 57 |
| M6h_2 | 44195924 | 6.63G | 39883322 | 5.83G | 90.24 | 98.34 | 95.01 | 56.99 |
| M6h_3 | 39142786 | 5.87G | 37204764 | 5.48G | 95.05 | 98.79 | 96.2 | 57.17 |
| M6h_1_Control | 37363618 | 5.60G | 35083328 | 5.16G | 93.9 | 98.95 | 96.62 | 57.27 |
| M6h_2_Control | 39387992 | 5.91G | 37359196 | 5.50G | 94.85 | 98.74 | 96.05 | 57.45 |
| M6h_3_Control | 38796178 | 5.82G | 36872738 | 5.43G | 95.04 | 98.73 | 96.02 | 57.03 |
| M12h_1 | 48021500 | 7.20G | 43694876 | 6.41G | 90.99 | 98.67 | 95.89 | 57.08 |
| M12h_2 | 43791094 | 6.57G | 40163860 | 5.88G | 91.72 | 98.22 | 94.68 | 57.11 |
| M12h_3 | 43684512 | 6.55G | 40633376 | 5.95G | 93.02 | 98.27 | 94.83 | 57.14 |
| M12h_1_Control | 37301424 | 5.60G | 35395946 | 5.22G | 94.89 | 98.85 | 96.32 | 57.26 |
| M12h_2_Control | 36930372 | 5.54G | 35176546 | 5.19G | 95.25 | 98.89 | 96.43 | 57.48 |
| M12h_3_Control | 36980502 | 5.55G | 33646950 | 4.69G | 90.99 | 99.13 | 97.21 | 55.53 |

**Table S3.** Summary of RNA-Seq data generated for control and treatment samples of *M. gypseum*
