# Supplementary material for: Antifungal activity and mechanism of Phoebe bournei wood essential oil against two dermatophytes
Source: Front Microbiol. 2025 Feb 7;16:1539918. doi: 10.3389/fmicb.2025.1539918 (PMC11842444; doi:10.3389/fmicb.2025.1539918)
Supplement: Supplementary file 12 [file Table_4.DOCX]

| Gene name | annotation |
| --- | --- |
| *ASCL1* | Long chain fatty acid CoA ligase 1 |
| *TPN1* | Vitamin B6 transporter |
| *PAL1* | Phenylalanine ammonia-lyase |
| *MUG72* | Meiotically up-regulated gene 72 protein |
| *GMT1* | GDP-mannose transporters |

**Table S4.** Functional annotation of module core genes
